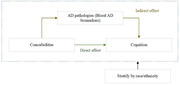# The effect of comorbidities on Alzheimer’s disease/cognition by race/ethnicity and the mediating role of AD pathologies measured by blood biomarkers

**DOI:** 10.1002/alz.089323

**Published:** 2025-01-09

**Authors:** Shanshan Wang, Uyen‐sa Nguyen, James Hall, Melissa Petersen, Sid E. O'Bryant, Rajesh Nandy

**Affiliations:** ^1^ University of North Texas Health Science Center, Fort Worth, TX USA

## Abstract

**Background:**

The study aims to assess the associations of comorbidities (hypertension, dyslipidemia, diabetes, chronic kidney disease [CKD], and depression) with mild cognitive impairment (MCI), Alzheimer’s dementia (AD dementia), and cognition by race/ethnicity; and to examine whether blood AD biomarkers mediate the associations.

**Method:**

We used data from the baseline and visit 2 of the Health & Aging Brain Study‐Health Disparities (HABS‐HD) study. 2057 participants were included in the cross‐sectional analyses, and 792 participants, who were cognitively normal at baseline and had follow‐up data, were included in the longitudinal analyses. Race/ethnicity was categorized into Latinos, non‐Hispanic Black, and non‐Hispanic Whites. Blood AD biomarkers included amyloid β40 (Aβ40), amyloid β42 (Aβ42), total tau, phosphorylated tau 181(p‐tau 181), and neurofilament light chain (NfL). Blood AD biomarker changes between the two visits were calculated. Global cognition was assessed by the Mini Mental State Examination (MMSE). Causal mediation analyses were used.

**Result:**

In the cross‐sectional analyses, the odds of AD dementia and MCI increased with higher levels of Geriatric Depression Scale (GDS) scores across all races/ethnicities. In the longitudinal analyses, those with CKD (β=‐0.431, 95% CI: ‐0.812, ‐0.049) and higher levels of GDS score (β=‐0.027, 95% CI: ‐0.050, ‐0.004) were negatively associated with global cognition at visit 2. While triglyceride (β=0.002, 95% CI: 0.0003, 0.003) and hemoglobin A1c (β=0.086, 95% CI: 0.002, 0.170) values were positively associated with global cognition at visit 2. Changes in Aβ40 and Aβ42 were found to be significantly associated with global cognition in both the overall sample and the Latino sample. The natural indirect effect of triglyceride on global cognition through changes in Aβ40 (β=0.0002, 95% CI: 0.00002, 0.0006) was found to be significant among the overall sample, with 15.80% of the association being mediated via changes in Aβ40.

**Conclusion:**

Our study is the first to examine the mediating role of blood AD biomarkers in associations of various comorbidities with AD and cognition by race/ethnicity. We found that changes in Aβ40 mediated the associations between triglyceride and global cognition, providing evidence that amyloid pathology might be the underlying mechanism.